# Effects of Dance Music on Motor Skills and Balance in Children: An Observational Cohort Study

**DOI:** 10.3390/children11091128

**Published:** 2024-09-18

**Authors:** Tadashi Ito, Hideshi Sugiura, Kentaro Natsume, Sho Narahara, Yoshifumi Sugimoto, Emi Matsuzawa, Hitomi Fujita, Yuji Ito, Kazunori Yamazaki, Natsuki Shimizu, Koji Noritake, Nobuhiko Ochi

**Affiliations:** 1 Three-Dimensional Motion Analysis Laboratory, Aichi Prefectural Mikawa Aoitori Medical and Rehabilitation Center for Developmental Disabilities, Okazaki 444-0002, Japan; 2 Department of Integrated Health Sciences, Graduate School of Medicine, Nagoya University, Nagoya 461-8673, Japan; 3 Department of Pediatrics, Aichi Prefectural Mikawa Aoitori Medical and Rehabilitation Center for Developmental Disabilities, Okazaki 444-0002, Japan; 4 Nagoya College of Medical Health & Sports, Nagoya 460-0008, Japan; 5 Department of Rehabilitation, Faculty of Health Sciences, Nihon Fukushi University, Handa 475-0012, Japan; 6 Department of Pediatrics, Nagoya University Graduate School of Medicine, Nagoya 466-8550, Japan; 7 Department of Information Systems, Faculty of Informatics, Daido University, Nagoya 457-0819, Japan; 8 School of Physical Therapy, Department of Health and Medical Care, Saitama Medical University, Saitama 350-0496, Japan; 9 Department of Orthopedic Surgery, Aichi Prefectural Mikawa Aoitori Medical and Rehabilitation Center for Developmental Disabilities, Okazaki 444-0002, Japan

**Keywords:** motor skills, dance, balance function, children, dance music

## Abstract

Background/Objectives: During the COVID-19 pandemic, children in Japan were restricted from sports and outdoor activities. Regular physical activity is essential for healthy growth and development in children, with recommendations advocating for activities such as strength training. However, the long-term effects of the restrictions have not been fully investigated. This study aimed to evaluate the effectiveness of an “exercise class”, specifically a dance program, designed to improve motor function in elementary school children affected by the coronavirus disease 2019 (COVID-19) pandemic, which may have compromised their motor function. Methods: The dance program was developed by a sports science teacher, a professional dancer, and a physical therapist, and it was evaluated in a community-based participatory study. Trained dance instructors conducted one-hour dance sessions in a medical center, held once a week for two months from June to August 2023. A physical therapist and dance instructors led the elementary school children. Outcome measures included grip strength, lower extremity muscle strength, back muscle strength, dynamic balance function during movement, physical activity time, and body fat percentages. Descriptive statistics were used for analysis. Results: Twenty-four children aged 6–9 years participated in the exercise class over a two-month period. Improvement was observed in grip strength, lower extremity muscle strength, and dynamic balance function during movement. However, physical activity time, body fat percentages, and back muscle strength did not change. Conclusions: The results indicated a significant improvement in motor skills. Dance is an inexpensive program that elementary school children can enjoy while improving their motor skills.

## 1. Introduction

Since December 2019, the coronavirus disease 2019 (COVID-19) has significantly affected people’s health worldwide. As of May 2023, COVID-19 has been classified as a category 5 infectious disease by the Japanese government. Quarantine measures have been lifted, allowing people to resume their normal activities. During the COVID-19 pandemic, children in Japan were restricted from sports and outdoor activities, resulting in fewer opportunities for physical activities and physical education in the school environment [1,2,3]. However, the long-term effects of these restrictions have not been fully investigated.

Physical inactivity can have long-term effects on the development and maintenance of motor skills. Prolonged restrictions on physical activity in primary schools may limit opportunities for adequate motor skill instruction. Evidence suggests a reduction in physical activity hours in children, leading to impaired balance function and muscle strength [4,5,6,7]. Consequently, physically inactive children may have lower levels of physical functioning and increased health risks later in life [6,8]. Therefore, post-pandemic efforts should ensure children have sufficient time for physical activity to prevent a decline in physical function. In addition, regular physical activity is essential for healthy growth and development in children, with recommendations advocating for activities such as strength training [9].

Recently, dance has emerged as a popular physical activity for children due to its potential to enhance performance [10]. It provides medium-to-high-intensity physical exercise and has proven to be effective in this regard [11]. Additionally, studies have reported improved motor skills after implementing physical and health education programs in physical education classes [12,13]. Moreover, demonstrative instructions were found to be more effective in teaching dance movements. However, the study did not observe any changes in motor function resulting from dance practice [14]. Nonetheless, a recent study highlighted dance as a viable option for traditional physical activities, offering physiological benefits to healthy individuals [9]. The study also emphasized the importance of policymakers seriously considering the implementation of dance programs in schools and the wider community [9]. Dance programs have been demonstrated to enhance balance function and muscle strength in children. Therefore, dance is a beneficial option for improving physical function in children post-COVID-19 pandemic [15,16,17]. Moreover, dance is a cost-effective, equipment-free, and easy-to-engage activity that can be performed at home [18]. In Japan, there is a growing concern regarding the decline in physical function due to a lack of exercise, particularly given the reduction in opportunities for outdoor play. To date, no studies in Japan have investigated the effectiveness of indoor dance programs for improving motor skills in elementary school children over a relatively short duration.

This study aimed to evaluate a short-term dance program designed to improve the physical function of local elementary school children in Okazaki, Aichi, Japan. Program participation, muscle strength, and balance function were the primary evaluation parameters. The study hypothesized and confirmed that the short-term program positively impacted the muscle strength and balance function of the children.

## 2. Materials and Methods

### 2.1. Dance Program Development

The dance program was created by implementing community participatory research methods consistent with previous studies [18,19], ensuring alignment with the requirements of the neighboring primary schools. Before initiating program development, a collaborative effort between academic and medical institutions involved children from Okazaki, Aichi Prefecture. These children underwent medical examinations and physical function evaluations before and during the COVID-19 pandemic to identify potential declines in physical function. The study revealed a tendency for decreased muscle strength and balance function. The program, designed to be performed in a safe indoor environment, was provided free of charge to participants. Based on the research conducted by Schroeder et al. [18], the program involved dance instructors, sports instructors, physical therapists, and pediatricians as key advisors. 

**Figure 1:**
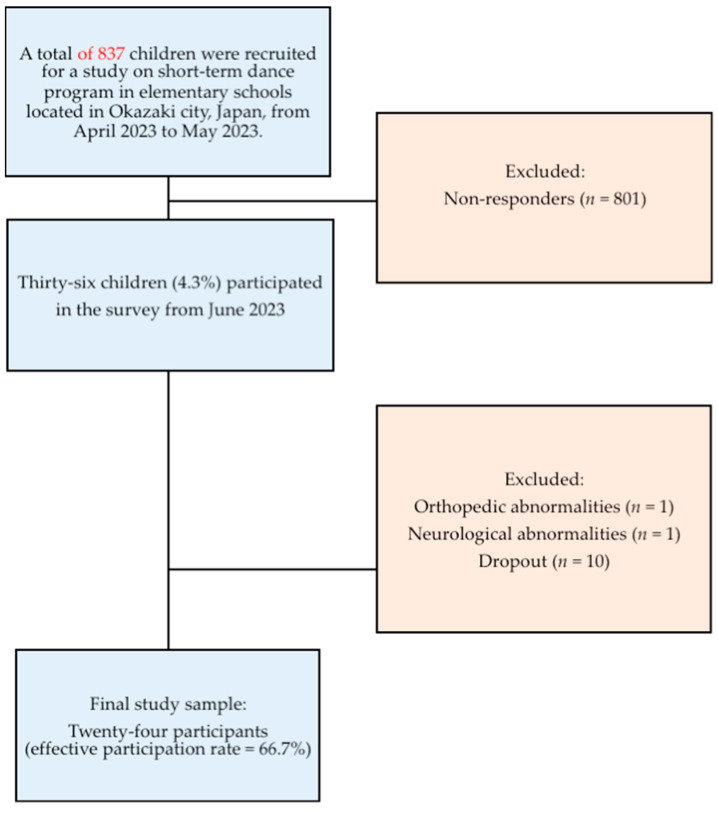
Flowchart of this prospective observational study.

Overall, 36 participants applied to participate in the dance program. Data were collected from the participants before the start of the class and once after its conclusion. Exclusion criteria included individuals with orthopedic, neurological, respiratory, ophthalmologic, or digestive disorders and those who had more than four absences from the dance program, which could have affected the results of the physical function assessment, rendering the data incomplete. Consequently, 12 of the 36 individuals were excluded, leaving a final sample of 24 in this study (Figure 1).

### 2.2. Dance Sessions

This study was conducted in accordance with the Declaration of Helsinki, and the protocol was approved by the Ethics Committee of the Aichi Prefectural Mikawa Aoitori Ethics Review Board (approval number: R4011). All participants and their legal guardians provided written informed consent and assent for participation in this study and for the publication of identifying information. Participants were recruited through flyers distributed in three nearby elementary schools in collaboration with teachers. The dance sessions, led by 12 instructors trained by the program’s developers, were held once a week for 8 weeks, from the 3rd week of June 2023 to the 2nd week of August 2023. The program was coordinated by a physical therapist and took place in the morning at a facility in Okazaki City, Aichi Prefecture. The dance consisted of a 1 h group session. The program aimed to enhance muscle strength and balance function through dance, featuring forward and cross-steps, and was performed to music (Supplementary Video Data S1).

### 2.3. Outcome Measurement

Data measurements were collected by physical therapy students who were trained and supervised by a pediatric physical therapist to assess the effectiveness of the dance classes. The training included interactive learning and hands-on practice in measuring height, weight, body fat percentage, and motor function.

#### 2.3.1. Grip Strength

Grip strength was measured using an adjustable Smedley hand-held dynamometer (GRIP-D, Takei Corporation, Niigata, Japan) as the average grip strength of both hands of the participants (kg). The dominant hand was measured first, followed by the non-dominant hand. Both hands’ grip strength was measured once in a seated position, with the participant rotating the shoulders abducted to the neutral position, elbows extended, and forearms and wrists in the neutral position [20]. Participants were instructed to tightly grip the dynamometer handle for 5 s [20,21]. One practice trial was allowed for learning purposes. The reliability of the grip strength measurement was reported to be superior when the elbow was fully extended [22].

#### 2.3.2. Standing Broad Jump

We measured the distance of participants’ standing broad jump while they were barefoot. Participants were instructed to stand with their feet slightly apart, behind the starting line, and to jump as far as possible with both feet simultaneously [23]. The distance was measured from the starting line to the closest heel upon landing. Participants were advised that resting at the landing point was not required. Each participant performed two jumps, and the longer distance was recorded. Participants were instructed to swing their hands when taking off, aiding propulsion [24]. Furthermore, oral support was provided to the participants during the jumps. The standing broad jump assesses instantaneous lower limb muscular strength [25].

#### 2.3.3. Back Muscle Strength

A digital back dynamometer (Back-D; Takei Ltd., Niigata, Japan) was used to measure back muscle strength [26,27]. Participants stood on a platform with their feet shoulder-width apart, and the length of the chain was adjusted by a research assistant according to height differences. Back muscle strength was measured based on the maximum isometric extension force of the trunk muscles while standing with the trunk flexed at 30° [26,27]. This position ensured that the force originated from the back muscles rather than the psoas or shoulder muscles, minimizing the risk of the participant falling backward. Measurements were taken twice, and the average value was recorded [28]. Back strength data were adjusted for body weight [27].

#### 2.3.4. Two-Step Test

To assess the dynamic balance function of the children in this study, they were directed to stand behind the starting line and take two steps as far apart as possible while aligning both feet [6]. The two-step test was assessed by calculating the ratio (in cm) of the distance (length in cm of the two steps) to the height (in cm) of the child [6]. Measurements were taken twice, and the highest value was selected for subsequent analysis.

#### 2.3.5. Physical Activity Time

The World Health Organization recommends at least 60 min of moderate and vigorous physical activity per day, at least 5 days per week, for children [29]. The number of hours per week spent engaging in moderate and vigorous physical activity was obtained from the guardians of the participants in question through the administration of a questionnaire. The total weekly hours of physical activity time were employed in the analysis. Parents were asked to report only time spent playing outside and indoor physical activity, excluding other extracurricular activities, club activities, and school physical education time that their children participated in. Time spent on dance practice at home was included in the physical activity.

#### 2.3.6. Body Fat Percentage

Body fat percentage was measured using a multifrequency bioelectric impedance analyzer (MC-780; Tanita, Tokyo, Japan). Participants followed standard positioning instructions, standing with the soles of their feet in contact with the anterior and posterior foot electrodes and grasping the hand electrodes with their palms and thumbs [5,6,7]. Skin-to-skin contact was avoided by keeping the arms extended and suspended in a natural standing position [5,6,7]. The assessments were performed by trained physical therapists or research assistants and were completed within 15 s [5,6,7]. The bioelectric impedance analyzer measured resistance at three electrical frequencies: 5, 50, and 250 kHz [5,6,7]. Using the data, the analyzer calculated the body fat percentage of the participants based on the relationship between the measured volume of electrical resistance and the conductor. Measurements were taken 2 h after meals in accordance with the provided instructions.

### 2.4. Sample Size

The sample size was determined using G*Power (Heinrich Heine University, Düsseldorf, Germany) [30,31]. Statistical power was set at 0.95, two-tailed alpha at 0.05, and effect size at large (d = 0.8). Based on these assumptions, the required sample size was 24.

### 2.5. Statistical Analysis

For statistical analysis, the data collected in this study were analyzed using SPSS version 28.0 (IBM Corp., Armonk, NY, USA). The Shapiro–Wilk test was used to verify the normal distribution of the variables, while the chi-squared goodness-of-fit test was used to compare differences in male-to-female ratios. Based on the results of the Shapiro–Wilk test, parametric data were analyzed using the paired-samples *t*-test and non-parametric data were analyzed using the Wilcoxon signed-rank test. In addition, a sub-analysis was conducted to compare the pre- and post-dance program outcomes separately for boys and girls. Two-sided *p*-values < 0.05 were considered statistically significant. Effect sizes of |r| < 0.1 were considered insignificant, |r| < 0.3 moderate, and |r| < 0.5 highly significant.

## 3. Results

Table 1 presents participant demographics, with a total of 24 participants over 2 months (*n* = 12 boys, *n* = 12 girls). Throughout this study, the chi-squared goodness-of-fit test did not demonstrate any significant differences related to gender (*p* = 1.000). Significant differences were observed in height (*p* < 0.0001), which increased throughout the program. However, there were no significant differences in weight, body mass index, or body fat percentages among the participants. Table 2 summarizes motor function improvements. Grip strength (*p* < 0.0001), standing broad jump (*p* = 0.024), and the two-step test (*p* = 0.001) showed improvement post-program. However, there was no change in physical activity time or back muscle strength. Of the participants, 45.8% attended eight sessions, while 54.2% attended fewer than eight sessions (88% attendance: *n* = 8, 75% attendance: *n* = 4, 63% attendance: *n* = 1). The results of the sub-analysis, as presented in Table 3 and Table 4, demonstrated that all measured outcomes improved in the boys following the implementation of the program. Conversely, for the girls, the sole outcome to demonstrate enhancement was grip strength.

## 4. Discussion

An evaluation of the free exercise class dance program has shown positive outcomes as well as areas for improvement. During the two-month evaluation period, the program was well-received by elementary school children, with 45.8% of participants attending all eight classes. We observed improvement in overall muscle strength, instantaneous muscle strength in the lower extremities, and dynamic balance function among participating children. However, no changes were observed in back muscle strength, physical activity time, or body fat percentage. The difference in frequency and duration of the dance program and the focus of the dances in this study on lower limb movements such as squatting, jumping, and stepping may explain why back strength did not improve as in a previous study [32]. Nevertheless, many of the findings of this study are consistent with those of previous studies [32,33]. However, it is worth noting that while previous studies have reported an increase in physical activity time with off-campus dance programs [32], this particular exercise class, conducted over a short period of time, did not yield such an increase. Gabriela et al. suggested that the lack of improvement in physical activity before and after the intervention could be attributed to the fact that medium- and high-intensity physical activities did not constitute 50% of the total class time in dance sessions [34]. Consequently, there may have been no corresponding increase in physical activity time in participants’ daily lives. Additionally, differences in the frequency and duration of the dance program compared to a previous study and the lack of instruction for participants to engage in independent dance practice at home might have contributed to the lack of improvement in body fat percentage [35]. Nonetheless, the observed improvements in muscle strength and balance function suggest that the dance program in this exercise class was likely of sufficient intensity for the children. One reason for this is that Japan only recently classified COVID-19 as a category 5 infectious disease in May 2023, indicating a significant lack of physical activity until the classification. Therefore, the short-term dance program presented in this study appears to have mitigated some of the adverse physical effects caused by long-term restrictions on outdoor activities [17]. 

The study confirmed that weekly exercise dance classes improved grip strength, standing broad jump performance, and two-step test results. Additionally, improvements in overall muscle strength, lower extremity muscle strength, and dynamic balance function were observed among children participating in the weekly 1 h dance sessions. The dance program, which included squatting, jumping, and stepping movements, likely contributed to these improvements. 

The significance of children’s participation in sports and dance activities outside of school to maintain healthy muscle strength has been previously highlighted [32]. Dance-based exercise classes, particularly when led by professionals, may be a suitable option for addressing children’s health and enhancing their motor skills. Motor development is associated with a child’s physical growth, as well as bone and muscle strength [9]. In a systematic review, children who participated in dance classes reported significantly improved motor development skills [9]. These findings suggest the positive impact of dance on total and instantaneous muscle strength of the lower extremities.

In this study, dynamic balance was assessed, and improvement was observed after the dance program. It has been suggested that dynamic balance may be improved by trunk stability training [17,36]. It is possible that the dance activities in this study positively affected some trunk muscles, consequently improving the children’s dynamic balance. However, further investigation with a control group may be needed to verify this hypothesis. Therefore, a short dance program may contribute to improved muscle strength and balance, which could be particularly beneficial for children who have been inactive.

Notably, the results of this study showed that a two-month dance program did not significantly improve physical activity time. Regardless, dance interventions are considered sustainable and flexible alternatives for increasing physical activity because of their high participation rate and ease of acceptance and implementation [9,37]. Furthermore, the program improves moderate-to-vigorous physical activity when delivered in a structured dance class in a dance studio [9,32,38]. The lack of improvement in physical activity time in this study may be because the home program was not encouraged. Moreover, it is important to exercise caution when interpreting the results of this study, as the questionnaire used was subjective and may have resulted in parents overestimating or underestimating their children’s physical activity time.

Additionally, the results of the sub-analysis demonstrated that the dance program was more efficacious for boys than for girls. The fact that the boys were more prone to enhance their total muscle strength, the instantaneous muscle strength of the lower extremities, back muscle strength, and their dynamic balance function was proposed as a possible explanation for this difference. Conversely, the findings indicated that while girls exhibited improvement in total muscle strength, their lower extremities’ instantaneous muscle strength and dynamic balance function demonstrated a reduced likelihood of improvement. These results suggest that boys and girls may respond differently even to the same dance program, with varying effects on the improvement of physical functions. 

This study suggests the possibility of developing short, cost-effective programs of as few as eight sessions that can be used to improve declining physical performance. Prior research has indicated that children’s motor abilities exhibit regional variations. They have also shown that fundamental motor competencies are comparable between genders and that motor skills tend to enhance with learning [39,40,41,42,43]. Thus, these programs could be integrated into physical education classes in local communities and schools. The present study’s findings may have been influenced by regional characteristics, given the selection of elementary school children from a specific region. Consequently, the efficacy of dance programs may not be universally applicable. Although this dance program focused on improving muscle strength and balance function, further research could target the improvement of other motor skills, such as motor coordination and sensation. Future research could focus on children diagnosed with developmental coordination disorders or those experiencing delayed motor development.

There are several limitations to this study that must be considered. First, this study was limited to children from grades 1 to 3 in elementary school only. Second, by focusing only on motor function, other important variables such as spatial, visual, and tactile perception, laterality, coordination, and attention were not assessed in this study. Thus, it is possible that some confounding effects related to these variables may not have been accounted for. Finally, because the present study did not establish a control group, the effects of the dance program can only be preliminarily considered.

Despite the importance of the findings from this observational study on dance programs, several limitations must be addressed. While the study included three elementary schools, providing an adequate sample size, all were located within the same community. Therefore, future studies may benefit from comparing children from different neighborhoods and backgrounds. In addition, a control group should be established to re-confirm the effectiveness of this dance program. However, given the observed improvements in muscle strength and balance skills with the involvement of a professional instructor, this short-term dance program could be recommended to individual schools and communities.

## 5. Conclusions

This observational study of a dance program showed that even a short-term program could significantly improve the motor skills of children who have been inactive due to the restrictive effects of COVID-19. With expert guidance, the program can be easily implemented even by children with no prior dance training. Therefore, it is recommended that dance programs be conducted in group sessions with a dance instructor.

However, further research is needed to fully understand the effects on children’s muscle strength and balance function and to develop optimal strategies to address them. Additionally, future studies should assess spatial cognitive function and coordinated movement in addition to motor function. 

## Figures and Tables

**Table 1:** Demographic characteristics of participants before and after the dance program (*n* = 24; 12 girls and 12 boys).

Variable	Before the Dance Program	After the Dance Program	*p*-Value	95% CI	Effect Size (r)
Age (years), median (range)	8 (6–9)	8 (6–9)	0.317	0.001–0.001	−0.2
Height (cm)	126.2 (0.9)	126.7 (7.5)	0.0001	−0.765–−0.260	0.7
Weight (kg), median (range)	23.3 (14.4–44.0)	23.7 (15.2–43.3)	0.061	0.001–0.500	−0.4
Body mass index (kg/m^2^), median (range)	14.7 (12.1–22.6)	14.6 (12.4–22.2)	0.530	−0.138–0.228	−0.1
Physical activity time (h/week), median (range)	2.3 (0.5–7.0)	2.3 (0.5–10.0)	0.576	−0.500–0.833	−0.1
Body fat percentage (%), median (range)	11.3 (6.6–29.6)	10.1 (5.3–28.4)	0.265	−0.900–0.250	−0.2

Data are presented as means (standard deviations) and medians (ranges). The *p*-value for height was calculated using the paired-samples *t*-test, and the other *p*-values were calculated using the Wilcoxon signed-rank test. CI, confidence interval.

**Table 2:** Participants’ physical functions before and after the dance program.

Variable	Before the Dance Program	After the Dance Program	*p*-Value	95% CI	Effect Size (r)
Grip strength (kg)	9.1 (2.5)	10.6 (2.8)	0.0001	−2.155–−1.029	0.8
Standing broad jump (cm)	119.0 (26.5)	125.3 (22.6)	0.024	−11.612–−0.888	0.5
Back muscle strength	1.0 (0.3)	1.1 (0.3)	0.115	−0.230–0.027	0.3
Two-step test, median (range)	1.6 (0.8–1.8)	1.7 (1.0–2.0)	0.001	0.077–0.221	−0.7

Data are presented as means (standard deviations) and medians (ranges). The *p*-values of the grip strength, standing broad jump, and back muscle strength were calculated using the paired-samples *t*-test, and the other *p*-values were calculated using the Wilcoxon signed-rank test. CI, confidence interval.

**Table 3:** Boys’ physical functions before and after the dance program.

Variable	Before the Dance Program	After the Dance Program	*p*-Value	95% CI	Effect Size (r)
Grip strength (kg)	10.0 (2.7)	12.1 (2.6)	0.001	−2.974–−1.334	0.9
Standing broad jump (cm)	125.3 (26.9)	132.9 (22.7)	0.041	−14.938–−0.395	0.6
Back muscle strength	1.0 (0.2)	1.2 (0.3)	0.016	−0.332–0.019	0.6
Two-step test, median (range)	1.5 (0.8–1.7)	1.7 (1.1–1.8)	0.002	0.092–0.294	−0.9

Data are presented as means (standard deviations) and medians (ranges). The *p*-values of the grip strength, standing broad jump, and back muscle strength were calculated using the paired-samples *t*-test, and the other *p*-values were calculated using the Wilcoxon signed-rank test. CI, confidence interval.

**Table 4:** Girls’ physical functions before and after the dance program.

Variable	Before the Dance Program	After the Dance Program	*p*-Value	95% CI	Effect Size (r)
Grip strength (kg)	8.1 (2.0)	9.2 (2.2)	0.011	−1.770–−0.288	0.7
Standing broad jump (cm)	112.8 (25.8)	117.7 (20.6)	0.263	−13.859–4.192	0.3
Back muscle strength	1.1 (0.3)	1.1 (0.3)	0.788	−0.248–0.193	0.1
Two-step test, median (range)	1.6 (0.9–1.8)	1.6 (1.0–2.0)	0.071	0.014–0.254	−0.5

Data are presented as means (standard deviations) and medians (ranges). The *p*-values of the grip strength, standing broad jump, and back muscle strength were calculated using the paired-samples *t*-test, and the other *p*-values were calculated using the Wilcoxon signed-rank test. CI, confidence interval.

## Data Availability

All relevant data are presented in this manuscript. All the data are available from the authors upon request.

## Supplementary Materials

The following supporting information can be downloaded at: https://www.mdpi.com/article/10.3390/children11091128/s1.
